# Dogs Exhibiting High Levels of Aggressive Reactivity Show Impaired Self-Control Abilities

**DOI:** 10.3389/fvets.2022.869068

**Published:** 2022-03-24

**Authors:** Elena Gobbo, Manja Zupan Šemrov

**Affiliations:** Department of Animal Science, Biotechnical Faculty, University of Ljubljana, Domžale, Slovenia

**Keywords:** dogs, police dogs, inhibitory control, delay of gratification, reversal learning, aggression

## Abstract

Inhibitory control describes a multitude of cognitive processes that prevents an impulsive response and enables a more appropriate behavior in a given situation. The ability to inhibit undesirable behaviors, such as aggression, is particularly important in dogs for safe and successful interspecific interaction and cooperation. The present study investigated the associations between two aspects of inhibitory control in dogs, self-control and cognitive inhibition, and the tendency to respond aggressively when provoked. Sixteen police and fourteen privately owned dogs of the same sex, breed group and similar age participated. Self-control, often described as impulsivity, was measured with an exchange paradigm themed the delay of gratification test, and cognitive inhibition with an object discrimination paradigm called the reversal learning test. Aggressive reactivity was assessed with a standardized aggression-eliciting behavior test. When comparing police and privately owned dogs, police dogs showed higher aggression levels and poorer self-control, while the two groups did not differ in cognitive inhibition. Regardless of the dog group, the main results indicated impairments in self-control in dogs with high levels of aggressive reactivity. Dogs showing biting behavior had worse self-control abilities compared to dogs with no signs of aggression. No association between cognitive inhibition and aggression was found. We conclude that self-control, measured as the ability to tolerate delayed rewards, appears to be an important aspect of inhibitory control involved in the tendency to respond aggressively, particularly in police dogs.

## Introduction

Aggression can be observed in a variety of species and can be defined as a behavior that inflicts or threatens physical or psychological harm (1). In dogs, it is generally expressed as aggressive biting behavior, by snapping or attacking, and aggressive threatening behavior, by growling, barking, and baring their teeth (2). Although it is one of the normal social behaviors of dogs (2), aggression represents one of the most dangerous and undesirable behaviors in certain contexts, especially when directed toward humans. The ability to respond non-aggressively facilitates interactions with humans and allows the development of relationships (3–5). Therefore, further understanding of aggression may be important for animal welfare, public safety, and improved dog-human cooperation.

The most objective way to assess aggression in dogs in a control environment is to use standardized behavioral tests known to assess aggressive reactivity (i.e., the tendency of dogs to respond aggressively), such as Socially Acceptable Behavior (SAB) test (6). The validation of the SAB test revealed that the behavior shown in the test is highly associated with the dogs' past and future behavior. Therefore, it is suitable to assess behavioral phenotypes by including dogs with different behavioral backgrounds. For example, the selection of dogs in the study presented in this manuscript was based on Haverbeke et al. (7), who found frequent aggression of military dogs in the SAB test and on our previous findings, which showed that privately owned but highly trained dogs rarely expressed aggression in the SAB test (8).

There is recent evidence showing that canine aggression is associated with a number of psychological and cognitive factors. For example, it may be associated with various dog and owner personality traits (9, 10), temperament (11), attachment styles (10), impulsivity (5), and cognitive impairment (12). Another cognitive mechanism proposed to play a role in aggression is inhibitory control, referred to as the ability to interrupt the execution of an immediately enticing but detrimental behavior in favor of a delayed but more rewarding behavior (13). Reduced inhibitory control ability has been reported to be associated with aggression and violence in human adults (1, 14) and children (15). Although it has been previously suggested that dogs have the ability to inhibit behaviors unwanted by their owners (4), there are large gaps in knowledge regarding the association between aggression and inhibitory control.

Inhibitory control in dogs is usually measured using simplified versions of tests developed for humans [e.g., (16)] and non-human primates [e.g., (17)]. Using different tests, both human (18) and canine (13, 19, 20) researchers found that the tests did not correlate with each other, but appeared to be context-specific. The lack of correlation suggests that the individual tests measure different aspects of ability, suggesting that inhibitory control is a collection of distinct cognitive processes rather than a unified mechanism (19, 21). Therefore, it is important that it is captured with multiple tests, each targeting different aspects of this ability. Three aspects of inhibitory control are commonly described in dogs: motor inhibition, self-control, and cognitive inhibition (19, 22). Self-control and cognitive inhibition are aspects known to be associated with human aggression (23, 24), but it is not known whether such an association exists in species such as dogs.

Self-control is the ability to tolerate a certain effort in order to obtain a better outcome [see (21) for a review], and it is proposed to be an important determinant of whether an individual overrides or responds to an urge to react aggressively (25). It is commonly measured using an exchange paradigm called the delay of gratification test, in which an individual must abstain from a less preferred reward and wait for a better but more delayed reward (26). It has been suggested that the ability to inhibit a prepotent response is evidence of better self-control because it leads to receiving more or a better quality reward (21). To our knowledge, the ability to tolerate delayed rewards has not yet been studied in the context of canine aggression, but studies in humans (24) and rats (27) have shown that aggressive individuals show less self-control.

Cognitive inhibition, on the other hand, is the ability to regulate the content of working memory by blocking information irrelevant to the task (28). It is often measured using an object discrimination paradigm called the reversal learning test, in which two stimuli change their reward contingencies after initial discrimination learning (29). The test measures flexibility in relearning object-reward contingencies, but also the ability to inhibit a learned response and avoid the previously rewarded option (19, 29). Again, this paradigm has not yet been used in the context of canine aggression, but impairments in reversal learning have been associated with aggression in humans (23).

We focused on the two aspects of inhibitory control, self-control and cognitive inhibition, and we aimed to investigate their association with aggressive reactivity in dogs, using a standardized behavioral test and two separate tests of inhibitory control. Based on studies in humans and rats, we predicted that dogs would show limited inhibitory control in both tests when characterized as more aggressive during aggression-eliciting stimuli. Compared to privately owned dogs, we predicted that police dogs would exhibit higher level of aggression and poorer cognitive performance, because outside of their working environment they often display impaired self-control (30).

## Materials and Methods

The Administration of the Republic of Slovenia for Food Safety, Veterinary Sector and Plant Protection approved the study (U34401-17/2020/10). All participants signed a consent form and were given the right to withdraw from the study at any time. We hereby confirm that the study was performed in accordance with the relevant guidelines and regulations.

### Animals

Thirty dogs (Supplementary Table 1) participated in the aggression and two inhibitory control tests. Included dogs had different aggression-related behavioral phenotypes, but comparable demographic characteristics. They were either privately owned and were highly trained or had various working functions (*n* = 14) or were police dogs at the process of training, not specialized in a particular working task (*n* = 16). Privately owned dogs lived at owner's home (*n* = 14), while the police dogs lived either at handler's home (*n* = 4), in kennel (*n* = 7) or the combination of the two (*n* = 5) (Supplementary Table 1). All dogs were male, between 12–36 months of age (mean age: 22.00 ± 6.65 months) and from the same classified breed group—sheepdogs (Fédération Cynologique Internationale) and except two privately owned dogs, all others were neutered. Similar age and breed of dogs mitigate the effect of age (31) and breed (32) on inhibitory control performance. Males were chosen because majority of police dogs in the country are males. From previous research it is known that male dogs have a higher probability of aggression than females (33), making them more suitable to study in the context of aggression.

### Procedure

The testing was conducted between July and October 2020 at the two different sites. Using the same equipment and procedures, police dogs were tested at the site of the Ministry of Interior of the Republic of Slovenia and privately owned dogs were tested at the Biotechnical Faculty of the University of Ljubljana. Due to police dogs availability and logistical limitations, mainly including the size and installation of the outdoor test area, all dogs first participated in the aggression test. About 2 weeks later, inhibitory control testing was performed in an empty indoor test room (5 × 6 m) unknown to the dogs. Following the procedure and set-up modified after Brucks et al. (19), self-control was measured with the delay of gratification test and cognitive inhibition was assessed with the reversal learning test. Due to limited availability of indoor space because of COVID-19 restrictions, both were administered on the same day. There was approximately half an hour rest period between tests and the owner/handler was allowed to walk the dog outside or freely interact with the dog inside (e.g., if the weather was bad). The order of testing was counterbalanced and randomized for all dogs. To control for fatigue and satiation, the order of testing, number of trials and quantity of food the dog received were noted down. None of the dogs had been previously trained for these specific tests. Immediately before the test, the owners/handlers were informed how to follow the experimenter's instructions, and the dogs were allowed to explore the room freely for 2 min. During the test, which was videotaped, only the owner/handler and a female experimenter (not the same person performing the aggression test) were present in the room. The owners/handlers were passive during the tests, except when instructed to release and call back their dog.

### Aggression Test

Aggressive reactivity was assessed using the SAB test (6). Dogs were subjected to 16 subtests containing stressors known to elicit aggression in dogs. Descriptions of the subtests are presented in the Table 1. The test was performed outdoors in an enclosed test area of 700 m^2^ (8). The owner/handler passively guided the dog on a leash during subtests 1–7 and 16 and was absent during subtests 8–15, when the dog was alone and attached with a fixed leash. Three female experimenters performed the test; the lead experimenter guided the owners/handlers through the test and the other two performed the subtests. Subtests were videotaped and aggression was scored using the scoring method developed by van der Borg et al. (34). For each subtest, aggression was scored on a 3-point scale, with 0 points awarded when there was no evidence of aggression, 1 point for threatening behavior (e.g., growling, baring teeth), and 2 points for attacking behavior (e.g., snapping, biting). The dogs were assigned into three categories, depending on the aggression level displayed; no aggression (received 0 point on all the subtests), only threatening behavior (received a score 1 on at least one of the subtests) or biting behavior (received a score 2 on at least one of the subtests).

**Table 1:** Descriptions of 16 socially acceptable behavior subtests from Gobbo and Zupan Šemrov (8).

**Subtest**	**Description**
1	The dog is approached by one tester and petted with an artificial hand
2	The dog is exposed to an unfamiliar visual stimuli (a blanked is pulled up and down)
3	The dog is exposed to an unfamiliar visual stimuli (sudden appearance of a cat on a sledge)
4	The dog is exposed to an unfamiliar sound (sudden activation of a horn)
5	The dog is exposed to an unfamiliar sound (sudden rattle of metal cans)
6	The dog is slowly approached and surrounded by three testers
7	The dog is rapidly approached and surrounded by three testers
8	The dog is approached by one tester with a dummy dog
9	The dog is slowly approached by one tester and petted using an artificial hand
10	The dog is exposed to an unfamiliar sound (a bell is rang in front of the dog)
11	The dog is exposed to an unfamiliar visual stimuli (an umbrella is rapidly opened and closed in front of the dog)
12	The dog is exposed to an unfamiliar visual stimuli (a life-sized doll, standing on top of a sledge is pulled in front of the dog)
13	The dog is approached by one tester and petted with a doll fixed on a pole
14	The dog is approached by one tester staring.
15	The dog is approached by the same tester as in subtest 14 and petted with an artificial hand
16	The dog is approached by the owner or handler and petted with a doll

### Delay of Gratification

The delay of gratification test, described in Brucks et al. (19), measured self-control as the ability to forgo eating an accessible but low-quality reward (LQR) and wait for an inaccessible but high-quality reward (HQR). The test consisted of three parts: food preference test, training trials, and test sessions. To determine an LQR and HQR for each dog, the food preference test was conducted.

Different types of LQR (e.g., dry food) and HQR (e.g., sausage) (Supplementary Table 1) were cut into pieces (~1.5 × 1.5 cm). Based on owner/handler reports of their dogs' food preferences, one piece of LQR and one piece of HQR were placed on two separate, identical plastic bowls (height: 10 cm, diameter: 15 cm). The experimenter, positioned in front of the dog simultaneously moved the bowls attached to a 1 m pole toward the dog held on a leash by the owner/handler so that the dog could sniff them. The bowls were moved laterally (~1 m equidistant from the dog) and the dog was released and allowed to choose a bowl (i.e., eat the reward). This procedure was repeated twelve times, alternating sides of the LQR and HQR reward. If the dog chose the reward with the same quality at least nine times, that reward was considered its HQR and the less preferred reward was considered its LQR. If the dog did not choose the same reward at least nine times, the food combination was changed and the procedure was repeated.

**Figure 1:**
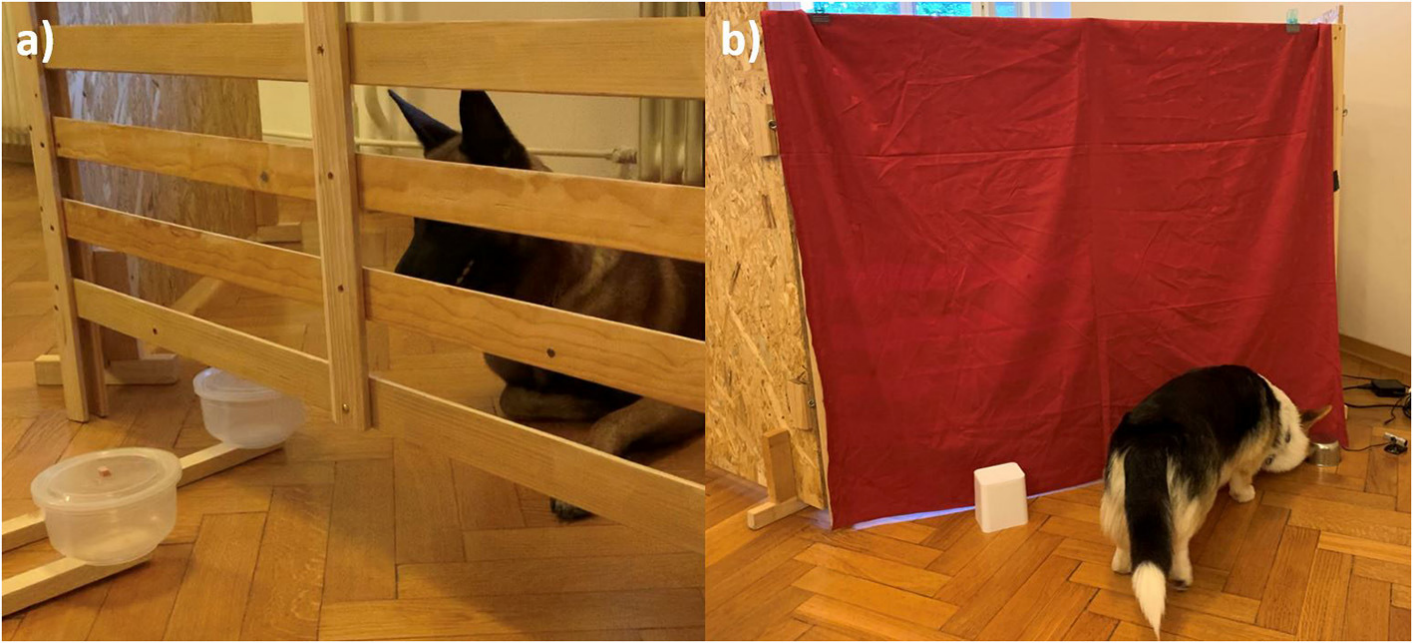
**(a)** Setup and bowls position for the delay of gratification test. The dog refrains from selecting the accessible LQR and waits for the HQR, which enters the enclosure after a certain delay. **(b)** Design and location of the bowls for the reversal learning test. The dog selected the metal bowl by approaching and touching it. Immediately thereafter, the experimenter, hidden behind the curtain, lifted the bowl to show whether it contained the reward.

After LQR and HQR were determined for each dog, the training trials followed. The owner/handler and dog entered a wooden test enclosure (2 m^2^), build out of three wooden frames (Figure 1a). The sides were covered in wood and the front part of the enclosure had an opening at the bottom through which two identical plastic bowls (the same shape as for food preference test) attached to a 1 m pole could be moved in and out. The experimenter, hidden behind a curtain, manipulated the two round plastic bowls, about 40 cm from the fence, and observed the dog via a webcam attached to the side of the enclosure. The movement of the two bowls was always as follows: Both bowls were pushed simultaneously toward the opening at the bottom of the fence so that both were visible but unreachable to the dog. Next, the bowl with the LQR entered the enclosure (until the whole bowl was inside, as shown in Figure 1a) and when the dog did not eat the reward, the bowl with the LQR was replaced by the bowl with the HQR after 2 s. Training was performed in order to familiarize the dog with the movement of the bowls and consisted of two types of trials: demonstration trials and test trials.

During the five demonstration trials, the owner/handler held the dog by the collar and prevented the dog from eating the immediately available LQR and released the dog when the LQR dish was withdrawn from the enclosure after a delay of 2 s and replaced with the HQR dish. During the test trials, the owner/handler remained passive and the dog was free to choose whether to eat the LQR immediately or wait for the HQR. The inter-trial interval was ~8 s. If the dog chose the HQR in at least three test trials, it proceeded to the next part of the test. If not, the training was repeated. If the dog did not reach the criterion within three trials, it did not progress to the last part of the test and its participation in the test was terminated.

The final part of the test, the test sessions, consisted of two parts, the demonstration sections and the main part of the test with increasing delay durations between LQR and HQR. To familiarize the dog with the delay duration, each test session started with the four demonstration trials where the owner/handler prevented the dog from eating the LQR after entering the enclosure and released the dog when the LQR was replaced by the HQR. The owner/handler then left the enclosure and hid behind the curtain, leaving the dog alone for the main part of the test. Beginning with a delay period of 2 s, ten trials were conducted and the dog's ability to wait (i.e., not eating LQR) for HQR was observed. When the dog reached criterion (waited for at least three trials), it proceeded to the next delay stage. The delay time was increased to 5 s, then to 10 s, and finally to 20 s in each successive test session. The maximum delay stage was selected based on Brucks et al. (35) finding 20 s delay is a specific turning point for dogs' success in this paradigm. If the dog did not reach the criterion, the test session was repeated with the same delay time. If the dog did not reach the criterion within three test sessions or successfully waited in the 20 s delay, the test was terminated. The number of successful trials during the final part of the test, as well as the maximum delay time achieved, was observed. For a more detailed description of the test, see Brucks et al. (19).

### Reversal Learning

Cognitive inhibition was measured as the ability to inhibit the previously learned response and shift the response to a new object-reward contingency, using the reversal learning test described in Brucks et al. (19). The test consisted of two phases; the acquisition phase and the reversal phase. The experimenter was hidden in a wooden enclosure covered with a curtain and observed the dog only via a webcam attached to the side of the enclosure. The owner/handler sat in a chair ~2 m from the enclosure and held the dog by the collar. Two different bowls were used for this test, one was smaller (height: 6 cm, diameter 8 cm), round and made of metal, the other was larger (height: 12 cm, diameter 10 cm), white and made of plastic (Figure 1b). Each phase began with four warm-up trials with the goal of having the dog associate a bowl with a reward (positive bowl). Half of the dogs were randomly assigned the metal bowl as the positive bowl, and the other half were assigned the white bowl. The experimenter took a piece of sausage with her fingers and, put her arm under the curtain, waved, and placed the reward on the floor. She then placed the assigned positive bowl on the reward and removed her arm. The owner/handler released the dog and the dog was allowed to approach the bowl. As soon as the dog touched the bowl, the experimenter lifted the bowl and the dog ate the sausage. The owner/handler called the dog back and the procedure was repeated for three more trials.

After the warm-up trials, the first session of the acquisition phase began. The experimenter placed both bowls in front of the curtain at the same time (Figure 1b), and the dog was released. When the dog chose the positive bowl, the experimenter lifted the bowl and the dog ate the reward. When the dog chose the other bowl (negative), the experimenter lifted the bowl so that the dog could see that there was no reward. Immediately thereafter, the experimenter quickly lifted the positive bowl so the dog could see where the reward was hidden without giving them the opportunity to eat the reward. Then the owner/handler called the dog back. Each session within the acquisition phase consisted of twelve trials with 10 s inter-trial interval and the position of the positive and negative bowl was alternated. If the dog identified the positive bowl in at least nine trials [*p* = 0.02; (19)] within a session, it reached criterion and moved on to the next phase. If not, the next session was repeated after a short break. If the dog did not reach criterion within three sessions, the test was terminated.

After the acquisition phase was completed, the reversal phase followed. Both the warm-up trials and the reversal phase were conducted using the same procedure as in the acquisition phase, with the previous negative bowl now containing the reward. The reversal phase involved only one session, consisting of 12 trials. The correct choices (selection of the bowl containing the reward) during the acquisition and reversal phases were calculated separately. According to Brucks et al. (19), the main inhibition measure represented the ratio between the number of correct choices during the last acquisition (session during the acquisition phase when the dog reached the criterion; LA) and the reversal phase (RP) (LA/RP ratio). The time from release to choice during each trial during LA and RP was also noted.

### Statistical Analysis

Frequencies (successful trials during the delay of gratification and correct choices during the RL) and continuous variables (time from release to choice during the RL) were coded using the Solomon coder (© 2019 by András Péter). Reliability coding was performed for 20% of the videos. The consistency between two coders for the continuous variable using an intraclass correlation coefficient was ICC > 0.88 and for the frequencies using Cohen's kappa was κ > 0.95.

Data were analyzed using SAS Software version 9.4 (Statistical Analysis Systems, SAS Institute, Cary, NC, USA). Normal distribution was determined using the Shapiro-Wilk test. With the exception of LA/RP ratio, the data distributions deviated significantly from the normal distribution, therefore non-parametric tests were used. Since all the dogs attending the test sessions in delay of gratification reached the maximum delay stage, the variable successful trials was treated as binary (dogs were either able or not able to delay gratification) and renamed to “success”. The Wilcoxon signed-rank test was employed to compare median scores (correct choice and time) during LA and RP in the reversal learning test and Mann-Whitney *U*-test for number of trials and quantity of food. Two-tailed Chi-square test and Cramer's V were used to examine the relationship between categorical variables (success, test order, group).

To assess differences in performance between police and privately owned dogs, non-parametric GLIMMIX procedure (Generalized Linear Model for Mixed procedure) was utilized for success, taking into account a Binomial distribution. For the purpose of multiple comparisons, a Studentised Maximum Modulus method was used. For LA/RP ratio and aggression level, MIXED procedure was utilized. For the purpose of multiple comparisons, Tukey-Kramer test was utilized. For all models, dog within group was used as a random effect and group (police or privately owned dogs) as fixed effect. The order of testing, number of trials and quantity of food were also considered as fixed effects, but very high correlations were found between these three variables (*p* < 0.001) in both of the groups and age had low variation, therefore these variables were not included in the final statistical models. Housing condition was also considered as fixed effect in the model, but due to the structure of the factor in the two groups, it was not included.

To evaluate the relationship between inhibitory control measures and the aggression test, a correlation analysis was performed using the Kendall rank correlation coefficient. Data were standardized using the *z*-transformation (36) to compare variables on the same scale. Eta Coefficient test was used to determine the strength of association between performance in the delay of gratification (success) and the reversal learning (LA/RP ratio). Statistical significance was accepted when *p* > 0.05.

## Results

One police dog did not participate in the delay of gratification and the reversal learning test due to anxiety. Another privately owned dog failed to learn the task in the reversal learning test. This means that 29 dogs participated in the delay of gratification test and 28 dogs participated in the reversal learning test. The order of tests was not associated with performance in the delay of gratification (Cramer's *V* = 0.11, *p* = 0.55), nor reversal learning (*r* = −0.09, *p* = 0.58). Police and privately owned dogs did not differ in the quantity of food received (*Z* = −0.02, *p* = 0.98) and the number of trials the dogs participated during the first test (*Z* = −0.02, *p* = 0.98). The association between success in the delay of gratification and LA/RP ratio the reversal learning was weak (η = 0.23).

### Delay of Gratification

During food preference test, the dogs needed between one and three sessions (mean: 1.63 ± 0.56), with 12 dogs (41.38%) having a preference during the first, 16 dogs (55.17%) during second and one dog (3.45%) during the third session. Out of 29 dogs, 17 dogs (58.62%) failed the training, 12 were police dogs. The 12 dogs (41.38%) that passed the training took an average of 2.38 ± 0.86 trials to reach the main part of the test. All 12 dogs successfully waited for the HQR during the delay phases and reached the maximum delay phase of 20 s. Throughout the test, the dogs waited between 24 and 42 trials for the HQR (median = 35.50 trials). Police dogs had significantly less success compared to privately owned dogs (*F* = 5.02, *p* = 0.033).

### Reversal Learning

Dogs made a higher number of correct choices during LA compared to RP and the time from release to choice was shorter during LA compared to RP (Table 2). On average, the dogs required 1.86 ± 0.69 sessions to reach the criterion for participation in the reversal phase. Police and privately owned dogs did not significantly differ in LA/RP ratio (*F* = 1.12, *p* = 0.30).

**Table 2:** Differences in performance during last acquisition and reversal phases in the reversal learning test.

**Variable**	**Phase**	**Median**	**Range**	***Z*-value**	***p*-value**
Time to make a choice (s)	Last acquisition	15.50	12.10–50.90	−4.64	**<0.001**
	Reversal phase	16.70	11.80–132.10		
Correct choices (number)	Last acquisition	10.00	9–11	−2.74	**0.006**
	Reversal phase	4.50	1–10		

*Bolded values show significant associations*.

### Association Between Inhibitory Control and Aggression

When provoked with aggression-eliciting stimuli in the SAB test, eleven dogs (36.67%) showed no aggression during the test and received a score of 0, seven dogs (23.33%) showed only threatening behavior, and 12 dogs (40%) showed biting behavior at least once during the test. Police dogs displayed a significantly higher aggression levels compared to privately owned dogs (*F* = 18.06, *p* < 0.001). The dogs with higher aggression level had less success during delay of gratification test and took less time to make a choice during LA (Table 3).

**Table 3:** Correlation between aggression level and *z*-transformed inhibitory control measures.

**Test**	**Measure**	**Correlation coefficient**	***p*-value**
Delay of gratification	Success	−0.44	**0.013**
Reversal learning	LA/RP ratio	−0.24	0.13
	Correct choices LA (number)	0.09	0.63
	Correct choices RP (number)	−0.26	0.10
	Time to make a choice in LA (s)	−0.36	**0.025**
	Time to make a choice in RP (s)	−0.26	0.10

*LA, last acquisition; RP, reversal phase. Bolded values show significant associations*.

Dogs showing distinct aggressive level differed in the success during delay of gratification test (χ^2^ = 6.41, *n* = 29, *p* = 0.041). Consideration of dogs that passed or failed training in the delay of gratification test revealed that of 17 dogs that failed training, 10 exhibited biting behavior, four exhibited threatening behavior, and three exhibited no aggression. Of the 12 dogs that passed the test, two showed biting behavior, three showed threatening behavior, and seven showed no aggression.

## Discussion

Focusing on two aspects of inhibitory control, self-control and cognitive inhibition, we investigated whether inhibitory behavior is associated with the occurrence of aggressive reactivity in dogs. In partial support our hypotheses, we found impairments in self-control, measured as poor performance in the delay of gratification task, but no effects of cognitive inhibition, measured with the reversal learning task, in highly aggressive individuals displaying biting behavior.

The results of the delay of gratification test need caution in interpretation due to the low variation in the performance. Because of that only failure or success were considered which may potentially limit the power of the results. Such performance was partially comparable to the results described in Brucks et al. (19). In both studies, more than half of the dogs were unable to pass the training and participate in the main part of the test, but our remaining dogs reached the maximum delay level compared to only 27 % in Brucks et al. (19). One of the explanations for this result could be found in the characteristics of the included dogs. We included mainly working or highly trained dogs, which, due to the nature of their work, are generally expected to have better cognitive performance compared to pet dogs (37) that participated in the other study. The other explanation could be the fact that we performed the test in 1 day, whereas in Brucks et al. (19) no more than three sessions were performed per day and the dogs had to continue the test on another day. Despite both tests being performed in 1 day, it appeared that order of testing, and consequently the number of trials a dog participated and quantity of food the dog received within the first test, did not affect the performance in the second cognitive test. Based on the self-depletion hypothesis, stating self-control in dogs relies on limited resources and once depleted, control of behavior becomes impaired (38), one could argue that our dogs would show impaired control of behavior following the delay of gratification test. However, our results showed that the participation in the delay of gratification did not affect further performance in the reversal learning test. Looking at the self-control results, dogs with the highest level of displayed aggression had the poorest performance in the delay of gratification tests. This is consistent with studies in humans (24) and rats (27) showing that individuals who have impaired self-control often exhibit aggression. It is well-established that self-control is one of the neuropsychological concepts included in a number of higher-order cognitive processes and is referred to as executive control (39). Executive control is involved in the self-regulation of emotions and actions, including aggression. Building on this, our behavioral data are also consistent with neuroscientific studies reporting impairments in the neural circuits underlying emotion regulation and executive control in aggressive dogs (40) and aggressive humans (41).

Another mechanism mediated by executive control is impulsivity (42), which is often described in the context of canine aggression (5, 43, 44). While the association between self-control, measured as performance in delay of gratification test, and aggression has not yet been assessed in dogs, it has been proposed that delay of gratification test is an index of impulsive behavior and that lack of self-control in dogs may also be referred to as impulsivity (5). In Fatjó et al. (43) it has been reported that impulsive dogs have reduced or absent warning signs before exhibiting aggression. In our study, we found that dogs that have difficulty in tolerating delayed rewards showed impulsivity, as only the dogs that showed biting differed from dogs without aggression in their performance in the delay of gratification. Similarly, executive control measures have been reported to be associated only with violent, but not non-violent crimes in humans (45). In addition, our results support the findings of Wright et al. (5) in which using questionnaire data reported by owners to assess impulsivity as a trait, it was reported that dogs that scored higher on the impulsivity scale were more likely to express aggression. Despite using a different methodological approach, the results are likely comparable as it has been reported that performance during the inhibitory control test is closely related to owners' subjective reports of the dog's impulsivity (46). In general, the association between impulsivity and aggression found in dogs mirrors the results of studies in humans (47), non-human primates (48) and rats (48) and seems to be consistent in a variety of mammalian species.

During the reversal learning test, results showed that several components of executive functions were measured. The dogs' performance declined and decision time increased during the reversal phase, confirming that cognitive inhibition was successfully measured. As our dogs frequently chose the previously rewarded option without being rewarded, performance showed inflexibility [i.e., impaired capacity for changing strategies; (49)] and compulsivity [i.e., repetition independent of feedback; (19, 50)]. In humans, impairments in reversal learning have been associated with aggression (23) and more compulsive individuals have been associated with more frequent outbursts of aggression (51). In contrast to the human literature and our prediction, we found no association between reversal learning performance and severity of displayed aggression in dogs; however, several problematic issues arise when directly compared with human studies that examined reversal learning. First, most human authors study impairment in reversal learning in the context of psychiatric disorders, such as attention-deficit/hyperactivity disorder, obsessive-compulsive disorder, and psychopathy, as these individuals are known to show increased aggression (52). The studies have often involved children (53) and thus compare well to dogs, as children and dogs share similar cognitive mechanisms (54, 55), interpreting results in psychiatric patients compared to dogs is difficult.

Second, impairments in reversal learning are associated with reactive aggression in people with psychiatric disorders, with higher vulnerability to experiencing frustration being the main factor contributing to reactive aggression (56). It is difficult to draw a similar conclusion from our data and specifically in dogs, as frustration in dogs has mainly been studied as a consequence of absence, inaccessibility, or decrease in value of food (57, 58) rather than as an underlying mechanism of aggression.

Since aggression level has only been associated with performance in the delay of gratification and not the reversal learning test, and the association between the two cognitive tests was weak, our finding further supports the context specificity of inhibitory control previously reported in dogs (13, 19, 20). Also, since most of highly trained non-police dogs performed well in the delay of gratification test, this finding supports the executive control hypothesis, stating that specific self-control training improves impulsivity in other contexts (59, 60). The lack of association between inhibition and aggression performances could be explained by the variation in the skills that the dogs had to possess in order to be successful during the test. This variation is described as task demand (13, 20), as each test has different demands and requires different regulatory and decision-making skills. For example, the mere visibility of the reward may influence performance during the test (61), as individuals have greater difficulty self-regulating themselves when rewards are fully visible than when they are hidden (62). Therefore, it may not be surprising that only performance in the delay of gratification test, where the rewards were constantly visible, and not in the reversal learning, where the rewards were hidden, was associated with a particular behavior.

As expected, police dogs exhibited higher levels of aggression, confirming previous findings that the majority of military dogs show aggression in the SAB test (7), in parallel with poorer self-control performance in delay of gratification. This is not surprising, as impaired impulse control in military dogs has already been demonstrated in other contexts, e.g., unwanted aggression outside their working domain (30). Our further results revealed no difference in cognitive inhibition between the groups. Compared to results in pet dogs (19), our dogs showed better cognitive inhibition in the form of more correct choices during reversal learning, confirming previous findings that trained working dogs have better cognitive inhibition compared to non-trained pet dogs (60). Since we had dogs with different working and non-working training backgrounds, we can assume that any type of high-level training may be associated with better reversal learning performance.

Despite a number of studies reporting no sex differences in inhibitory control in dogs (13, 19, 22), a recent study (63) found that female dogs displayed better inhibitory control, making investigation of sex differences an interesting aspect for future research. In our study, the fact that the aggression test was performed first followed by two cognitive test on the same day may present a serious limitation. Further replication with an improved experimental design is advisable. Notwithstanding this limitation, we believe the current study can be used as a foundation for further research, as we were, to our knowledge, the first to investigate whether different aspects of inhibitory control play a role in the occurrence of aggressive reactivity in dogs. Although no association was found between cognitive inhibition and aggression, it appears that self-control was the aspect of inhibition associated with the dog's tendency to respond aggressively when provoked. Dogs that were able to inhibit impulsive behavior in the delay of gratification showed less or no aggression, demonstrating the association between impulsivity and behavioral inhibition. Including only one dog breed, our finding may be difficult to generalize to entire dog population. We believe that further research is needed regarding impulsivity and aggression for several reasons. First, aggressive dogs, especially those that show aggression without warning signs, can be a serious problem for many dog owners and others involved (64). Second, impulsivity is highly consistent over time (65), the ability to characterize impulsive behaviors that may lead to aggression at an early age may not only be important scientifically, but may also benefit the general population of dog owners.

## Data Availability Statement

The raw data supporting the conclusions of this article will be made available by the authors, without undue reservation.

## Ethics Statement

The animal study was reviewed and approved by Administration of the Republic of Slovenia for Food Safety, Veterinary Sector and Plant Protection (U34401-17/2020/10). Written informed consent was obtained from the owners for the participation of their animals in this study.

## Author Contributions

EG and MZ: conceptualization, formal analysis, and writing—review and editing. EG: methodology, investigation, data curation, and writing—original draft preparation. MZ: supervision. Both authors read and approved the final manuscript.

## Conflict of Interest

The authors declare that the research was conducted in the absence of any commercial or financial relationships that could be construed as a potential conflict of interest.

## Publisher's Note

All claims expressed in this article are solely those of the authors and do not necessarily represent those of their affiliated organizations, or those of the publisher, the editors and the reviewers. Any product that may be evaluated in this article, or claim that may be made by its manufacturer, is not guaranteed or endorsed by the publisher.
