# Supplementary material for: Dogs Exhibiting High Levels of Aggressive Reactivity Show Impaired Self-Control Abilities
Source: Front Vet Sci. 2022 Mar 24;9:869068. doi: 10.3389/fvets.2022.869068 (PMC8987203; doi:10.3389/fvets.2022.869068)
Supplement: Supplementary file 1 [file Table_1.docx]

Supplementary Material

# Supplementary Figures and Tables

Supplementary Table S1. Demographic data of the dogs.

| No. | Age | Breed | Training | Housing | LQR | HQR |
| --- | --- | --- | --- | --- | --- | --- |
| 1 | 24 | Border Collie | Non-working training | Home | Cornflakes | Sausage |
| 2 | 20 | Border Collie | Non-working training | Home | Cornflakes | Sausage |
| 3 | 29 | Belgian Shepherd | Search and rescue | Home | Cornflakes | Sausage |
| 4 | 14 | Border Collie | Non-working training | Home | Dry food | Sausage |
| 5 | 24 | Border Collie | Non-working training | Home | Dry food | Cheese |
| 6 | 28 | Australian Shepherd | Search and rescue | Home | Sausage | Dry food |
| 7 | 15 | Pembroke Welsh Corgi | Non-working training | Home | Cornflakes | Sausage |
| 8 | 18 | Rough Collie | Non-working training | Home | Dry food | Sausage |
| 9 | 19 | Swiss Shepherd | Non-working training | Home | Sausage | Dry food |
| 10 | 25 | Belgian Shepherd | Obedience | Home | Dry food | Sausage |
| 11 | 36 | Belgian Shepherd | Obedience | Home | Dry food | Sausage |
| 12 | 24 | Australian Shepherd | Herding | Home^1^ | Cornflakes | Sausage |
| 13 | 36 | Australian Shepherd | Herding | Home^1^ | Cornflakes | Sausage |
| 14 | 36 | Border Collie | Non-working training | Home | Cornflakes | Sausage |
| 15 | 27 | German Shepherd | Police | Home | Dry food | Sausage |
| 16 | 27 | German Shepherd | Police | Mixed | Cornflakes | Sausage |
| 17 | 26 | German Shepherd | Police | Mixed | Dry food | Sausage |
| 18 | 15 | German Shepherd | Police | Kennel |  |  |
| 19 | 26 | German Shepherd | Police | Home | Cornflakes | Sausage |
| 20 | 21 | German Shepherd | Police | Mixed | Cornflakes | Sausage |
| 21 | 21 | Belgian Shepherd | Police | Home | Dry | Sausage |
| 22 | 21 | Belgian Shepherd | Police | Home | Cornflakes | Sausage |
| 23 | 19 | Belgian Shepherd | Police | Mixed | Cornflakes | Sausage |
| 24 | 19 | Belgian Shepherd | Police | Mixed | Cornflakes | Sausage |
| 25 | 15 | German Shepherd | Police | Kennel | Cornflakes | Sausage |
| 26 | 15 | German Shepherd | Police | Kennel | Cornflakes | Sausage |
| 27 | 15 | German Shepherd | Police | Kennel | Dry | Sausage |
| 28 | 15 | German Shepherd | Police | Kennel | Dry | Sausage |
| 29 | 15 | German Shepherd | Police | Kennel | Cornflakes | Sausage |
| 30 | 15 | German Shepherd | Police | Kennel | Dry | Sausage |

LQR = low quality reward, HQR = high quality reward, Home^1 =^ outdoor living, Mixed = living at handler’s home and in kennel
